# The role of personality traits, sociocultural factors, and body dissatisfaction in anorexia readiness syndrome in women

**DOI:** 10.1186/s40337-021-00410-y

**Published:** 2021-04-17

**Authors:** Karolina Rymarczyk

**Affiliations:** Institute of Psychology, Cardinal Stefan Wyszyński University in Warsaw, Warsaw, Poland

**Keywords:** Neuroticism, Big five dimensions, Sociocultural factors, Body dissatisfaction, Anorexia readiness syndrome

## Abstract

**Background:**

The mass media promote certain standards of physical attractiveness. The media coverage, in interaction with body dissatisfaction and personality traits, may intensify specified behaviors in women, that should help them to obtain an ideal body image, e.g., excessive concentration on body image, weight control, increase in physical activity. The intensification of these behaviors can develop anorexia readiness syndrome (ARS) in women. The paper presents a study on the role of the Five-Factor Model personality traits (neuroticism, extraversion, agreeableness, conscientiousness, and intellect/openness), sociocultural factors (internalization, sociocultural pressure, information seeking), and body dissatisfaction in anorexia readiness syndrome.

**Methods:**

The study involved 1533 Polish women aged 18–36 (*M* = 22.51, *SD* = 2.41). The participants completed the online version of the set of questionnaires. The link to the study was shared in social media groups. Personality dimensions were measured with the BFI, sociocultural factors were evaluated by means of the SATAQ-3, the degree of body dissatisfaction was assessed with the BIQ, while ARS was measured using five self-reported items referring to specific behaviors from TIAE.

**Results:**

Hierarchical multiple regression analysis revealed internalization, sociocultural pressure, and body dissatisfaction as significant predictors of ARS. While neuroticism was correlated with ARS, it lost its predictive value after entering body dissatisfaction in the regression model.

**Conclusions:**

The factors associated with ARS were (1) neuroticism among personality traits, (2) internalization and pressure from sociocultural norms among sociocultural attitudes, and (3) body dissatisfaction. The key finding is the absence of statistical significance for neuroticism in predicting ARS after including body dissatisfaction. In future research, the group of men and patients with anorexia nervosa can be included, and the age range can be extended to include younger people. The catalog of potential ARS predictors may be expanded, which can help to explain the role of neuroticism in ARS.

## Plain English summary

Mass culture conveys a lot of information that women should use to fit the specific canon of beauty regarding the female body. Media body image standards can be absorbed by many women who may feel the pressure of having to conform to these standards. Adopting and adhering to the sociocultural ideals of beauty contributes to the development of body dissatisfaction as well as anorexia readiness syndrome (ARS) that can even lead to eating disorders under certain non-favorable circumstances in the long term. The aim of the study was to examine the contributing factors of ARS – thoughts, emotions and behaviors that may lead to diagnosable anorexia nervosa (AN). The study involved 1533 Polish women from a nonclinical population. Based on a review of the literature, possible determinants of ARS were selected: personality traits, sociocultural factors and degree of body dissatisfaction. Analyses confirmed that internalization and pressure from sociocultural norms (two sociocultural factors), and body dissatisfaction may help predict ARS. Neuroticism (personality trait) was also related to ARS. Knowledge of which factors may contribute to ARS may help in understanding how AN develops and what treatments may help prevent the illness from developing. In future research, it is also worth conducting the study on a group of patients with eating disorders and a group of men. Research on a more diverse group will help to understand the determinants of ARS.

## Background

Mass media promote specified norms of attractiveness and the ideal female body and women can engage in certain behaviors, which in their opinion could help them to achieve this ideal body [1]. Such behaviors may be attributable to comparing oneself to unrealistic ideals while depreciating one’s attractiveness, and include: a greater interest in nutrition, calorie counting, dieting, weight control methods, increased physical activity, excessive focus on body image, emotional lability related to eating and body perception, the desire to control one’s body dimensions and weight, high competitiveness and perfectionism, as well as the need for control (cf. [2–4]). The above symptoms were classified by Ziółkowska [4] as elements of anorexia readiness syndrome (ARS) defined as “a set of indicators in the cognitive, emotional, and behavioral spheres of functioning giving rise to suspicion of abnormality in meeting one’s nutritional needs and in one’s attitude to one’s body” (p. 37).

Substantial research on ARS has been devoted to the extent of this phenomenon among adolescent and adult people. Factors that may lead to body dissatisfaction and, in the long term, to eating disorders, include sociocultural factors (cf. [2, 5, 6]) and personality traits (cf. [4, 7, 8]). However, in previous investigations those factors were examined separately. The present study focused on sociocultural factors and personality traits jointly in order to determine significant ARS predictors.

### Personality determinants of ARS

One of the personality models used in body image research is the Five-Factor Model [9], which consists of neuroticism, extraversion, agreeableness, conscientiousness, and intellect/openness to experience [9–11]. Research into the Big Five [9] has revealed that various personality traits have been significant in explaining body dissatisfaction, and especially neuroticism was relevant [12, 13] that: (1) was associated with perceiving one’s own body as larger than it is objectively over time [8]; (2) predicted body image dissatisfaction in men and women [12]; (3) predicted investment in body image and self-consciousness of appearance [14]; (4) was linked to actual-ideal weight discrepancy [15]; (5) was useful in explaining emotions related to depression caused by ideal self-discrepancies [16]; (6) was associated with eating disorders and low self-esteem [17]; (7) was a significant risk factor for eating disorder symptoms [18].

The trait of neuroticism (vs. emotional stability) comprises emotional instability, oversensitivity, the tendency to experience negative emotions, adjustment and resilience to stress [9]. Due to the tendency to experience negative emotions and dissatisfaction, neuroticism may be linked to negative body image (cf. [15]). Therefore, it would be worthwhile to examine the role of neuroticism in the development of ARS before the onset of a major eating disorder. Indeed, neuroticism appears to be a trait of special importance to disturbed body image, and ultimately to eating disorders (cf. [7, 12, 18]).

### Sociocultural factors as determinants of ARS

Body image perception in women is determined by various factors, that could labeled as sociocultural factors, and include: the assimilation of media influence and the internalization of the media body ideal [13, 19], the family environment [4, 20, 21], peer pressure among youth [22, 23], and cultural influence [24].

Thompson and collaborators [25], who termed sociocultural factors attitudes, distinguished: (1) internalization; (2) pressures from sociocultural norms; (3) seeking information about body image. The attitudes adopted by people are affected by the mass media, which promote certain norms of physical attractiveness (cf. [25, 26]). In conjunction with some physical characteristics (e.g., BMI) and psychological factors (e.g., ideal self-discrepancies, personality traits), media plays an important role in one’s body image (cf. [1, 27]).

The internalization of the thin ideal contributes to postponing the satisfaction of basic needs (e.g., food) and lowers the awareness of one’s own body in women [1]. The body ideal present in culture may affect body perception leading to objectification [28] and an instrumental attitude (using the body for attaining certain goals and benefits). According to the study of Młożniak and Schier [29], objectified body perceptions tend to be more severe in those who described their bodies as, e.g., a machine, instrument, or object [29].

The study of Izydorczyk and Rybicka-Klimczyk [1] showed that the mass media constitute an important source of information about beauty ideals for women. The subjects experienced pressure from sociocultural norms in connection to the female body image standards promoted by the media and internalized them to a significant extent. In their paper on ARS, Chytra-Gędek and Kobierecka [5] found that the key assessment criterion for women was being slim, which was associated with good looks, popularity, as well as being successful and attractive to men. Based on the responses given in the study, in the group of examined women, 6.5% of them were found to exhibit ARS (mostly in the dimension of attributing high value to body slimness) [5].

Sociocultural factors play a role not only in eliciting body dissatisfaction, but also in the development of eating disorders [6, 30–32], which may follow from persistent dissatisfaction with one’s looks, a critical evaluation of one’s appearance, and vulnerability to external influence (e.g., the mass media). Therefore, these factors should be examined in terms of their predictive value for ARS.

### Body dissatisfaction as a determinant of ARS

Body image may be defined as an internalized belief about one’s appearance [33], including convictions about how one is perceived by others and the associated emotions (cf. [34]). The cognitive dimension of body image consists of individuals’ thoughts, beliefs, and cognitive patterns about their appearance. A negative perception may imply a lack of acceptance leading to adverse emotional states, excessive weight control, a tendency for perfectionism, and internalization of attractiveness standards [4, 5, 35, 36]. The cognitive sources of distorted body image are also investigated by neuroscience. Its affective element may be associated with changes in the prefrontal cortex, insula, and amygdala, with the perception component being linked to changes in the parietal lobe (cf. [37]).

Body dissatisfaction and negative emotional states often result from comparisons with the ideal [38–40] that is frequently and conspicuously presented in the mass media [41, 42]. Abnormal body image perceptions may be also attributable to depressive symptoms or major depression [43]. Rzeszutek and Schier [43] reported that young adults with depressive symptoms tended to be more critical of their bodies. Body dissatisfaction may result from discrepancies between real perceptions of one’s body and of the body presented in the media and entails a higher risk of eating disorders [39]. Hagman et al. [44] showed that patients with anorexia nervosa (AN) had a higher level of body dissatisfaction than healthy women and overestimated their body size more.

Therefore, if body dissatisfaction is associated with the risk of developing eating disorders, it seems reasonable to explore its role in predicting ARS.

### Present study

The present study explored possible determinants of ARS among the variables described above, identified in the literature as the most important predictors of ARS. One can expect that (a) personality traits and especially neuroticism (cf. [18]), (b) sociocultural factors, and especially internalization and pressure (cf. [3]), and (c) body dissatisfaction (cf. [2]) are positively related to ARS. Moreover, a question has been asked which of these variables will significantly increase the prediction of ARS. According to the Five-Factor Theory, in the first step personality traits (basic tendencies) were included in the analysis and in the next steps sociocultural factors and body dissatisfaction (characteristic adaptations) were added [45].

Considering that the image of the human body in the mass media, which has major implications for the way women perceive their own bodies [41, 42, 46], and numerous findings pointing to the significant role of neuroticism in body image perception, hierarchical multiple regression analysis was conducted to find out whether the aforementioned categories of variables can be used to predict ARS.

## Methods

### Participants

The study encompassed 1533 Polish women aged 18 to 36 years (*M =* 22.51*, SD =* 2.41), and was administered online using the University Online Survey System. The participants were recruited from groups in social media where the link to the study was shared. There were student groups and groups related to women’s activity. Participants’ anonymity was fully ensured, and the study did not include personal questions. The inclusion criterion was age - 18 years or more.

Among the participants, 52% had secondary education, 47.1% had higher education, with the remaining 0.9% having primary or vocational education. As many as 79.6% of the women were city dwellers, while 20.4% lived in rural areas. The medium BMI level was 22.10 (13.63–51.27). Most of the examined women (*n* = 1079) were of a normal weight (18.5–24.9), 203 women were underweight (below 18.5), and 169 women were pre-obese (25.0–29.9). In the BMI group of obesity, 58 women were obesity class I (30.0–34.9), 14 women were obesity class II (35.0–39.9), and only 6 women were obesity class III (above 40). Women also completed the Photographic Figure Rating Scale to assess the most closely resembled their own bodies (real) and the body that they would like to possess (ideal). To assess the direction of the real-ideal discrepancy, we used the Modified Contour Drawing Figure Rating Scale [47], which included nine line-drawings of women’s bodies with covered faces. The real-ideal discrepancy showed that 1203 women would like to be thinner, 114 women would like to be fatter, and in 216 women there were no differences. Although the group of women who would like to be thinner was large, most of the women were still of a normal weight (BMI 18.5–24.9).

### Measures

#### Measurement of personality traits

Personality traits were measured using the *Big Five Inventory* (BFI) [10], which consists of 44 items describing various behaviors. BFI evaluates five personality dimensions: extraversion, agreeableness, conscientiousness, neuroticism, and openness/intellect [11] using a five-point scale ranging from 1 (*strongly disagree*) to 5 (*strongly agree*). The Cronbach’s alpha of the constituent trait scales ranged from 0.70 to 0.84.

#### Measurement of sociocultural factors

Sociocultural factors were measured using the *Sociocultural Attitudes Towards Appearance Questionnaire-*3 (SATAQ-3) [25]. The Polish version of the questionnaire prepared by Izydorczyk and Rybicka-Klimczyk [1, 26] consists of 24 items forming three scales (as opposed to four scales in the original): (1) internalization evaluating the degree of comparing oneself to and assimilating sociocultural norms concerning attractive body image standards, and especially those promoted by the mass media (nine items, e.g., “I wish I looked like the models in music videos”); (2) pressure from sociocultural norms assessing the strength of that pressure exerted by the mass media (seven items, e.g., “I’ve felt pressure from TV or magazines to lose weight”); (3) seeking information about body image measuring the frequency of seeking that information in the mass media (eight items, e.g., “TV commercials are an important source of information about fashion and “being attractive”). Responses were given on a five-point scale ranging from 1 (*definitely disagree*) to 5 (*definitely agree*), with the Cronbach’s alpha of the various scales being between 0.80 and 0.91. Since the scales revealed significant intercorrelations of more than 0.54, the overall score was used in the study (STQ). The overall score was supported by the scree plot on all 24 items which clearly shows a single factor that explains 42.44% of the variance of all variables while the second explains only 8.58%. The correlations of individual scales with the overall score exceeded 0.80. A parallel analysis was run on four scales and indicates a one-factor solution (first two eigenvalues from sample correlation matrix: 2.146, 0.908; first two average eigenvalues from parallel analysis: 1.056, 1.016).

#### Measurement of body dissatisfaction

The degree of body dissatisfaction was measured using the *Body Image Questionnaire* (BIQ-40) developed by Głębocka [39] containing 40 items grouped into four subscales (1) the cognition-emotions subscale evaluates one’s opinions about one’s appearance as well as the emotions experienced in relation to it (16 items, e.g., “I think my hips are too big”); (2) the behavior subscale consists of items referring to a healthy lifestyle (five items with reversed response scale, e.g., “I enjoy active leisure”); (3) the criticism from others subscale measures the subjective degree of acceptance of the woman by the people around her (six items, e.g., “I often hear critical comments about my looks”); (4) the pretty-ugly stereotype measures the degree of internalization of the prevalent standards of beauty (13 items, e.g., “Slim women are more attractive to men”).

The overall score indicates the degree of dissatisfaction with one’s body image. Using the overall score was supported by the scree plot on items which clearly shows a single factor that explains 32.09% of the variance of all variables while the second explains only 9.82%. A parallel analysis was run on three scales and indicates a one-factor solution (first two eigenvalues from sample correlation matrix: 2.171, 0.459; first two average eigenvalues from parallel analysis: 1.041, 1.001). Responses were given on a five-point scale from 1 (*definitely disagree*) to 5 (*definitely agree*) with the reliability for the overall score being Cronbach’s alpha = 0.95, and that for the constituent subscales ranging from 0.81 to 0.95.

#### Measurement of ARS

ARS was measured using behavioral indicators from the questionnaire for *Testing Individual Approach to Eating* (TIAE) [4]. Five items were selected due to their specific content: (1) “I’ve fasted, dieted, restricted food”; (2) “I’ve taken purgatives”; (3) “I’m angry when I eat too much”; (4) “I’ve taken weight-loss drugs/appetite suppressants; (5) “I’m trying to cut down on fat and carbohydrates”. Categorical confirmatory factor analysis, in which the selected items formed a latent factor, fitted the data well: *df* = 5; *χ*^*2*^ = 53.3; CFI = 0.976; RMSEA = 0.079 [0.061–0.099]. Responses were given on a binary scale from 0 (*no*) to 1 (*yes*). The total score is the sum of five behavioral items selected from the categorical confirmatory factor analysis. The factor loadings of the items ranged from 0.53 to 0.86. Cronbach’s alpha for the five selected items was 0.66.

### Statistical analyses

There were no missing data for the self-report questionnaires because the answer to each question was required. In the first step, correlation analysis was conducted to compare associations between the studied variables: personality traits, sociocultural factors, body dissatisfaction, and ARS. Subsequently, hierarchical multiple regression was performed because we wanted to see how including subsequent variables changes the ARS prediction in the model [48]. The regression model was verified in three steps: after entering each variable, it was checked whether the percentage of explained variance increased. In this way, four variables were entered in three steps (called “blocks” in SPSS software): (1) personality traits; (2) sociocultural factors; and (3) body dissatisfaction. In each step we introduced variables assumed to be related to ARS based on theoretical reasoning and additional separate analysis. The variables had been included in this specific order based on Five-Factor Theory from basic tendencies (personality traits) by characteristic adaptations (sociocultural factors) to self-concept (body dissatisfaction) ([45], p. 192). It is especially important taking into account that personality traits are rather stable and hardly change while the other factors are much more prone to changing in the interventions.

After introducing each set of variables, it was checked whether the resulting model explained more variance and which variables were significant in predicting ARS. In particular, it was investigated whether neuroticism, which was significantly associated with body dissatisfaction, would maintain its significance after the introduction of other variables associated with body image.

## Results

### Descriptive statistics

Descriptive statistics for the scales used in the study are given in Table 1.

**Table 1:** Descriptive statistics of the questionnaires used in the study

		Min	Max	Mean	Standard deviation	Skewness	Kurtosis
BFI-44	Extraversion	1.13	5.00	2.93	0.78	– 0.03	– 0.63
	Agreeableness	1.00	5.00	3.46	0.59	– 0.20	0.01
	Conscientiousness	1.33	5.00	3.47	0.67	– 0.18	– 0.25
	Neuroticism	1.25	5.00	3.47	0.76	– 0.27	– 0.34
	Openness	1.40	5.00	3.71	0.63	– 0.33	– 0.09
SATAQ-3	Internalization	1.00	4.89	3.12	0.82	– 0.28	– 0.81
	Pressure from sociocultural norms	1.00	5.00	3.16	1.08	– 0.31	– 0.89
	Information seeking	1.00	5.00	2.53	0.83	0.17	– 0.47
BIQ-40	Cognition-emotions	1.00	5.00	2.80	1.04	0.17	– 1.08
	Behaviors	1.00	5.00	2.80	0.90	0.08	– 0.61
	Criticism from others	1.00	4.83	1.96	0.74	1.06	1.00
	Pretty-ugly stereotype	1.00	5.00	3.61	0.65	– 0.32	0.37
	Overall score	4.56	18.54	11.16	2.43	0.29	– 0.27
TIAE	ARS	0.00	1.00	0.46	0.19	0.15	– 0.51

Table 2 presents descriptive statistics (*M* and *SD*), intercorrelations, and correlations between personality traits, sociocultural factors, body dissatisfaction, and ARS.

**Table 2:** Descriptive statistics, intercorrelations, and correlations between the scales

	BFI-44					SATAQ-3				BIQ-40					TIAE
	E	A	C	N	O	IN	PSN	IS	STQ	BD	C-E	BEH	CRI	P-U	ARS
E	–														
A	0.25**	–													
C	0.16**	0.18**	–												
N	- 0.37**	- 0.32**	- 0.29**	–											
O	0.31**	0.14**	0.16**	- 0.12**	–										
IN	- 0.11**	- 0.10**	- 0.18**	0.29**	**0.00**	–									
PSN	- 0.09**	- 0.07**	- 0.13**	0.28**	- 0.04	0.60**	–								
IS	- 0.04	- 0.05	- 0.07**	0.17**	- 0.07*	0.61**	0.54**	–							
STQ	- 0.10**	- 0.09**	- 0.15**	0.29**	- 0.04	0.85**	0.87**	0.83**	–						
BID	- 0.28**	- 0.18**	- 0.31**	0.43**	- 0.17**	0.48**	0.50**	0.33**	0.52**	–					
C-E	- 0.16**	- 0.10**	- 0.20**	0.35**	- 0.08**	0.52**	0.55**	0.33**	0.55**	0.87**	–				
BEH	- 0.29**	- 0.15**	- 0.31**	0.27**	- 0.25**	0.12**	0.14**	0.07**	0.14**	0.60**	0.27**	–			
CRI	- 0.23**	- 0.16**	- 0.23**	0.35**	- 0.12**	0.29**	0.27**	0.21**	0.30**	0.74**	0.58**	0.25**	–		
P-U	- 0.11**	- 0.14**	- 0.13**	0.27**	- 0.02	0.46**	0.49**	0.37**	0.52**	0.68**	0.60**	0.12**	0.35**	–	
ARS	- 0.15**	- 0.16**	- 0.12**	0.35**	0.03	0.55**	0.50**	0.41**	0.57**	0.62**	0.72**	0.04	0.46**	0.58**	–
*M*	2.93	3.46	3.47	3.47	3.71	3.12	3.16	2.53	8.81	11.16	2.80	2.80	1.96	3.61	0.46
*SD*	0.78	0.59	0.67	0.76	0.63	0.82	1.08	0.83	2.32	2.43	1.04	0.90	0.74	0.65	0.19

Notes: **statistical significance at *p* < 0.01; * statistical significance at *p* < 0.05; *n* = 1533

BFI-44: *E* Extraversion, *A* Agreeableness, *C* Conscientiousness, *N* Neuroticism, *O* Openness; SATAQ-3: *IN* Internalization, *PSN* Pressure from sociocultural norms, *IS* Information seeking, *STQ* overall SATAQ-3 score; BIQ-40: *BD* Body dissatisfaction, overall score, *C-E* cognition-emotion, *BEH* behavior, *CRI* criticism from others, *P-U* pretty-ugly stereotype; TIAE: *ARS* anorexia readiness syndrome, *M* mean, *SD* standard deviation

Considering the Big Five dimensions, it should be noted that neuroticism was most strongly correlated with body dissatisfaction (*r* = 0.43) and ARS (*r* = 0.35). In turn, the overall score calculated for the SATAQ-3 (STQ) was more closely associated with body dissatisfaction (*r* = 0.52) and ARS (*r* = 0.57) than any of its constituent scales, although the correlation between internalization and ARS was of a similar level (*r* = 0.55). A strong correlation was also found between ARS and body dissatisfaction (*r* = 0.62). Interestingly, there was no relationship between the Big Five openness and internalization (*r* = 0.00).

These correlations indicate that neuroticism is associated with the degree of body dissatisfaction, internalization, and ARS. In women, the tendency to adopt abnormal attitudes to eating and body image under stress was found to be strongly predicted by the degree of body dissatisfaction, and even more so by the opinions and emotions about one’s appearance as well as the degree of internalization, that is, comparing oneself to and assimilating sociocultural norms concerning ideal body standards, especially those promoted by the mass media.

### Regression analysis

The next step involved analysis of the hierarchical multiple regression model containing significant predictors of the dependent variable. The assumptions for the correlation and the linear regression were verified before conducting the analyses. The normal distribution has been confirmed based on values of skewness and kurtosis. Skewness and kurtosis statistics revealed that the results had a normal distribution, as they were in the standard deviation range of <− 1; + 1>. All predictor variables and the outcome variable were quantitative. The assumptions of multicollinearity (Variance inflation factor, VIF = 1.5605) and independent errors (Durbin-Watson test, DW = 1.726) were also satisfied. All the predictor variables were not correlated very highly (correlations of above 0.80 or 0.90) [48].

In order to predict ARS, four variables were introduced in three blocks: (1) personality traits, (2) sociocultural factors, and (3) body dissatisfaction. First, a linear regression analysis of personality traits showed that only neuroticism significantly predicted ARS (neuroticism: β = 0.19, *p* < 0.01; extraversion: β = − 0.004, *p* > 0.01; intellect/openness: β = 0.06, *p* > 0.01; agreeableness: β = − 0.03, *p* > 0.01; conscientiousness: β = − 0.02, *p* > 0.01). Thus, neuroticism was entered in hierarchical multiple regression analysis in the first block. The second block included two variables: internalization and pressure from sociocultural norms (sociocultural attitudes to body image) because in the separate regression, the third sociocultural factor (information seeking) was an insignificant predictor of ARS. The variable entered in the third block was body dissatisfaction, which was associated with emotions related to one’s appearance. Three models with a good fit to the data were obtained, with analysis of variance being statistically significant for each of them (Table 3).

**Table 3:** Summary of hierarchical multiple regression analysis

Model	Corrected *R*^2^	Standard error	*R*^2^ change	*F*	*df1*	*df2*	Significance
1	0.04	1.38	0.04	61.86	1	1531	0.000
2	0.19	1.27	0.15	143.47	2	1529	0.000
3	0.27	1.20	0.08	168.41	1	1528	0.000

Notes: Model 1: neuroticism; Model 2: internalization, pressure from sociocultural norms; Model 3: body dissatisfaction

Model 1 with one predictor (neuroticism) explained as little as 4% of the variance in ARS. Model 2 with three predictors (neuroticism, internalization, and pressure from sociocultural norms) explained 19% of the variance. Finally, Model 3, containing four predictors (neuroticism, internalization, pressure from sociocultural norms, and body dissatisfaction), explained 27% of the variance (Table 4).

**Table 4:** Regression coefficients in the hierarchical multiple regression model

Model		*b*	Standard error	Beta	*t*	Significance
1	Constant	0.40	0.17	–	2.40	0.02
	Neuroticism	0.37	0.05	0.20	7.87	0.00
2	Constant	- 0.92	0.17	–	- 5.32	0.00
	Neuroticism	0.12	0.05	0.07	2.75	0.01
	Internalization	0.38	0.05	0.22	7.58	0.00
	Pressure from sociocultural norms	0.31	0.04	0.24	8.20	0.00
3	Constant	- 1.74	0.18	–	- 9.84	0.00
	Neuroticism	- 0.06	0.05	- 0.03	- 1.38	0.17
	Internalization	0.24	0.05	0.14	5.00	0.00
	Pressure from sociocultural norms	0.17	0.04	0.13	4.67	0.00
	Body dissatisfaction	0.21	0.01	0.36	13.00	0.00

As can be seen, Model 3 with four predictors explained the largest percentage of the variance. It is worth noting that after the introduction of the variable “body dissatisfaction” neuroticism lost statistical significance: β = − 0.03; *p* > 0.01.

As far as the research question is concerned, analysis showed that the only personality trait significant to body image, i.e., neuroticism was not retained in the hierarchical multiple regression model after entering other variables, equally relevant to body image. Thus, the final set of predictors significantly explaining ARS comprised internalization, pressure from sociocultural norms, and body dissatisfaction.

## Discussion

The obtained results confirmed the adopted hypotheses about correlations between the Big Five factors, internationalization, pressure from sociocultural norms, body dissatisfaction, and ARS. Hierarchical multiple regression analysis revealed three variables that significantly predicted ARS, i.e., internalization, pressure from sociocultural norms, and body dissatisfaction.

Among the Big Five factors, only neuroticism exhibited the highest significant correlation with body dissatisfaction, which is consistent with the findings of Swami et al. [15], where neuroticism was significantly positively associated with body image in women (the higher scores on the scale indicate greater actual-ideal weight discrepancy) and significantly negatively associated with body appreciation (the higher scores on the scale reflect more positive body appreciation). In the study on male subjects, Benford and Swami [49] found a positive correlation between neuroticism and drive for muscularity; while body appreciation was negatively related to neuroticism and positively to extraversion.

The presented study also analyzed sociocultural factors using the SATAQ-3. It was found that the higher the degree of internalization, the higher the extent of anorectic behaviors. A similar correlation, albeit somewhat weaker, was obtained for pressure from sociocultural norms. Thus, internalization was shown to be a significant predictor of ARS. Internalizing the beauty standards promoted by the mass media, women engage in behaviors aimed at attaining the ideal, such as the tendency to give in to mass culture, excessive concentration on the body image, comparing oneself to paragons of beauty, and emotional lability related to body image. However, when such behaviors intensify, women do not necessarily come closer to the ideal, but rather expose themselves to the risk of eating disorders (cf. [4]), which may be associated with sociocultural factors, as indicated by Murnen i Smolak [31] in a meta-analysis or relations between distorted body image and disordered eating. Anorectic behaviors may be also affected by one’s opinion of, and emotions elicited by, one’s appearance, as well as general body dissatisfaction, as shown by the high correlation identified in this study.

Hierarchical multiple regression analysis revealed three variables that significantly predicted ARS, i.e., internalization, pressure from sociocultural norms, and body dissatisfaction. Initially, the model also included neuroticism, but in the third step, after entering body dissatisfaction, neuroticism lost its statistical significance despite it being correlated with body dissatisfaction. In a similar study, Swami et al. [13] found that body dissatisfaction was significantly negatively correlated with extraversion, emotional stability, and openness, but regression analysis showed that the Big Five dimensions explained only a small proportion of the variance in body dissatisfaction. The authors suggested that the internalization of media influence may be the dominant predictor of body dissatisfaction, even more so than personality traits. Swami et al. [13] also showed that openness was not significantly associated with internalization-general (− 0.08), as in this article (0.00). Openness, as a personality trait, describes ideas, creativity, curiosity, and aesthetics [45]. Internalization is assimilating sociocultural norms (concerning attractive body image standards) [25], which is rarely considered as openness and following ideas, but rather as a demand associated with the achievement of certain aims (attractive body image). In psychopathology, internalizing disorders have been predicted by neuroticism and low conscientiousness [10]. Martin et al. [50] also reported that body dissatisfaction was significantly associated with neuroticism and with thin-ideal internalization (internalization of societal ideals of attractiveness). In turn, Reshadat et al. [51], who explored the relationship between personality traits and self-efficacy in weight control with unhealthy eating behaviors and attitudes, reported a significant negative correlation between such behaviors and neuroticism and psychoticism. As a result, neuroticism and psychoticism may be used to predict unhealthy eating behaviors. Finally, in the mentioned study of MacNeill et al. [12] disordered eating patterns were predicted by high neuroticism and extraversion as well as low conscientiousness in women, but not in men. Body image dissatisfaction was predicted by neuroticism in women and by neuroticism and low conscientiousness in men. The significant predictor of body image dissatisfaction was also BMI in both groups [12]. Those findings were partially confirmed in the present study, albeit only with neuroticism in models no. 1 and 2, which contained neuroticism, but not in model 3, from which that trait was eliminated. Thus, it should be considered whether the role of the general neurotic tendency is not in fact overestimated. According to model no. 3, internalization, pressure from sociocultural norms, and body dissatisfaction have greater explanatory power for ARS. Under the circumstances, neuroticism in particular and personality, in general, are no longer significantly associated with body image perceptions.

### Limitations

The main limitations of the study are: (1) the focus on only women; (2) a cross-sectional design; (3) one country study. Future studies need some extensions to overcome these limitations. Future research could include not only women but also men. The study could be conducted on healthy people and patients diagnosed with eating disorders. A cross-cultural study could also provide substantial relations between personality, social factors, and body dissatisfaction in different groups. Future research could also include longitudinal studies and its results could verify the preventive and screening role of ARS.

## Conclusions

The study examined the importance of personality traits, sociocultural attitudes, and body dissatisfaction on ARS in women. The factors most closely associated with ARS were (1) neuroticism among personality traits, (2) internalization and pressure from sociocultural norms among sociocultural factors, and (3) body dissatisfaction. The key finding is the absence of statistical significance for neuroticism in predicting ARS, even though that trait was strongly correlated with body image. The significant variables predicting ARS were internalization, pressure from sociocultural norms, and body dissatisfaction.

### Implications

The knowledge of the predictors of ARS can be useful for the prevention of anorexia nervosa. The results in this article emphasize the important role of internalization and pressure from sociocultural norms and body dissatisfaction in the prediction of ARS. Neuroticism lost its role in predicting ARS after introducing these factors and this can be seen as an outcome that makes the prevention possible. Neuroticism is a stable, consistent, and enduring personality characteristic, determined biologically and therefore not changeable while sociocultural factors and body dissatisfaction could be changed. Thus, decreasing neuroticism in prevention or intervention can be impossible but increasing internalization and pressure from sociocultural norms or body dissatisfaction can be feasible. If those results will be confirmed in subsequent studies, anorexic behaviors (ARS symptoms) can be screened and preventively assessed in order to counteract the development of anorexia nervosa in the long term by focusing on changing these factors.

## Data Availability

The datasets generated and analysed during the current study are available in the OSF repository: https://osf.io/b3s48/.

## Abbreviations

ARS: Anorexia readiness syndrome
BFI: Big Five Inventory
BMI: Body Mass Index
BIQ-40: Body Image Questionnaire
SATAQ-3: Sociocultural Attitudes Towards Appearance Questionnaire-3
TIAE: Testing Individual Approach to Eating
